# Corrosion Susceptibility and Allergy Potential of Austenitic Stainless Steels

**DOI:** 10.3390/ma13184187

**Published:** 2020-09-21

**Authors:** Lucien Reclaru, Lavinia Cosmina Ardelean

**Affiliations:** 1 Scientific Independent Consultant Biomaterials and Medical Devices, 103 Paul-Vouga, 2074 Marin-Neuchâtel, Switzerland; lreclaru@gmail.com; 2 Department of Technology of Materials and Devices in Dental Medicine, “Victor Babes” University of Medicine and Pharmacy Timisoara, 2 Eftimie Murgu sq, 300041 Timisoara, Romania

**Keywords:** austenitic steels, general (uniform) corrosion, pitting corrosion, crevice corrosion, intergranular corrosion, galvanic couplings, nickel release, contact with skin, medical devices, watchmaking

## Abstract

Although called stainless steels, austenitic steels are sensitive to localized corrosion, namely pitting, crevice, and intergranular form. Seventeen grades of steel were tested for localized corrosion. Steels were also tested in general corrosion and in galvanic couplings (steels–precious alloys) used in watchmaking applications. The evaluations have been carried out in accordance with the ASTM standards which specifically concern the forms of corrosion namely, general (B117-97, salt fog test), pitting (G48-11, FeCl_3_), crevice (F746-87) and intergranular (A262-15, Strauss chemical test and G108-94, Electrochemical potentiodynamic reactivation test). All tests revealed sensitivity to corrosion. We have noticed that the transverse face is clearly more sensitive than the longitudinal face, in the direction of rolling process. The same conclusion has been drawn from the tests of nickel release. It should be pointed out that, despite the fact that the grade of steel is in conformity with the classification standards, the behavior is very different from one manufacturer to another, due to parameters dependent on the production process, such as casting volume, alloying additions, and deoxidizing agents. The quantities of nickel released are related to the operations involved in the manufacturing process. Heat treatments reduce the quantities of nickel released. The surface state has little influence on the release. The hardening procedures increase the quantities of nickel released. The quantities of released nickel are influenced by the inclusionary state and the existence of the secondary phases in the steel structure. Another aspect is related to the strong dispersion of results concerning nickel release and corrosion behavior of raw materials.

## 1. Introduction

Corrosion represents an important factor in the design and selection of metals and alloys for different purposes, as various corrosion mechanisms can lead to failure [1,2]. Corrosion resistance is an important criterion for selecting materials used, because the cost of their degradation due to corrosion and the associated environmental impact are quite substantial [3]. Like all metals, stainless steels can undergo chemical corrosion over time [4,5,6].

Corrosion manifests in different forms and depends on a multitude of physico-chemical factors (chemical composition and microstructure of the alloy, temperature, pH, chemical composition of the environment) and mechanical factors (stresses, friction) [7]. The relationship between corrosion rate and grain size has been revealed in numerous studies [8,9,10,11]. Its importance lies in the fact that this parameter can be tailored by the producers [8,9,10,11].

The austenitic steels belong to the stainless steels family and are being characterized by high Ni_eq_ and Cr_eq_ [12,13]. Over time, the chemical composition, mechanical properties, resistance to corrosion, machinability and polish ability of the austenitic steels have evolved considerably, and new production processes have been developed by the steel manufacturers [14]. Each chemical element in their composition plays an important role in their properties [15], including corrosion resistance, and can be substantially modified by adding certain elements as Cu, Ti, Nb, Al, Si and Ca. Generally, the composition of austenitic stainless steels is adjusted to meet service requirements in various corrosive environments [8]. The corrosion sensitivity of austenitic steels mainly takes the form of pitting, crevice and intergranular type [16].

An important aspect which concerns the austenitic steels is the release of nickel in contact with the skin. The role of nickel in the biological response to alloys is significant with regard to toxicology and biological performance. The current trend is to eliminate nickel from alloys for medical applications. However, this needs a careful evaluation since no compromise is acceptable concerning the mechanical properties, corrosion resistance or any other possible undesirable consequences due to the substitution of nickel [7,17].

Nickel allergy is the most widespread of all contact allergies. In the European population, the prevalence of nickel allergy is of 10%–15% of adult females and 1%–3% of adult males [18,19,20,21,22]. Of nickel-sensitive people in the general population, 30% develop hand eczema. Teenagers and young adults tend to have a higher prevalence due to frequent body piercing.

In Europe, for objects containing nickel, intended for permanent contact with skin, Directive 94/27/EC imposed a ban if the rate of nickel release exceeds 0.5 µg/cm^2^·week. The subsequent Directive 2004/96/EC: “Piercing in the Human Body” specifies that the limit rate of nickel release, for these cases, is 0.2 µg/cm^2^·week [23,24,25].

The aim of this study is to evaluate the sensitivity, under the same conditions, of 17 austenitic steels of the 304, 316 and 904 series, for uniform, pitting, crevice and galvanic corrosion. Our interest was also to assess the behavior differences of the transverse surface of the samples compared to the longitudinal one.

## 2. Materials and Methods

Table 1 shows the composition of the austenitic stainless steels (exception #16 and #17) which were used to prepare the samples for the corrosion evaluation tests.

According to the classification of Fontana [26], the evaluation of their behavior in uniform, pitting, crevice, intergranular corrosion was presented, as well as in galvanic couplings.

### 2.1. Salt Fog Test

To illustrate uniform corrosion, four austenitic steels—#2-1.4427, #3-1.4435/316L, #4-316L/1.4435Ugim and #5-1.4435/316LVal—were tested by using the salt fog test, according to ASTM B117-97 [27].

The samples were of cylindrical shape—10 mm diameter, 5 cm long. To create a reference state, as-received samples were first annealed and recrystallized at 1050 °C. The thermal treatment was carried out in a continuous industrial oven under hydrogen protection with gas cooling.

Half of the annealed cylinders were cold-worked to diameter 7.7 mm. The purpose of this cold-working operation was to increase the samples’ sensitivity to corrosion [28,29]. The entire surface of the samples was “mirror” polished. The test was carried out over a period of 12 days, 6 days in 5% NaCl medium and 6 days in artificial sweat medium diluted 40 times (Table 2). Artificial sweat medium ISO 3160-2 has the following composition: NaCl 0.5 g/L; NH_4_Cl 0.4375 g/L; Acetic Acid 0.063 g/L; Urea 0.125 g/L; Lactic Acid 0.375 g/L; NaOH solid, necessary quantity to induce a pH of 4.7.

The test in question was an adaptation of the ASTM B117-97 standard [27] which is in current use for metallic objects in contact with the skin (ISO 3160-2). Three samples were used for each state, making a total of 48 samples.

### 2.2. Pitting Corrosion

Steels #1–#17 (Table 1) were tested for pitting corrosion. The test samples, in the form of wires, 10 mm diameter, 5 cm long, were fixed in a resin and “mirror” polished.

The tests were carried out in the most commonly used electrolytes: FeCl_3_ and NaCl, and artificial sweat, according to ISO 3160-2:2015 [30]. The different electrolytes were used to verify the observations. The chemical compositions of the media used and the experimental conditions are presented in Table 3.

The immersion times were therefore different: 15 days for FeCl_3_ 0.1 M, 26 days for NaCl 0.5 M and 30 days for artificial sweat (Table 3), because of the difference in aggressiveness of the media (chemical composition, concentration) towards the evaluated materials [34].

### 2.3. Crevice Corrosion

**Figure 1:**
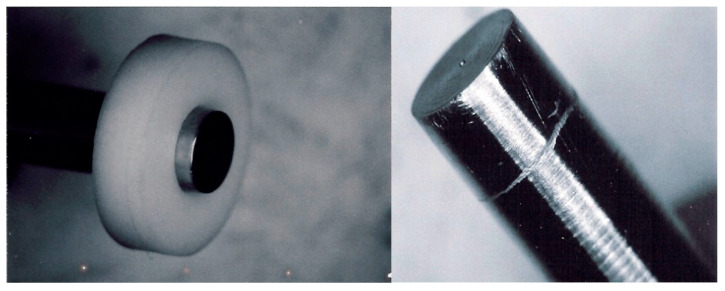
Specific assembly for the crevice corrosion test. Crevice corrosion aspect of the test sample.

The crevice corrosion test was carried out according to ASTM F746-87 [35]. The samples, of cylindrical shape—6.35 mm diameter, 5 cm long—obtained by machining of 10-mm-diameter steel profiles, “mirror” polished, were embedded in a polytetrafluoroethylene (PTFE) collar at one of the extremities (Figure 1). Both transverse and longitudinal surfaces were subjected to the corrosion test.

**Figure 2:**
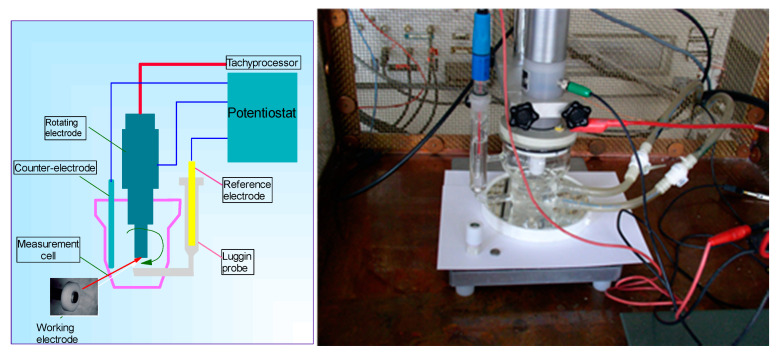
The electrochemical assembly and the electrochemical measuring cell.

The sample was mounted in the rotating electrode of the measuring cell (Figure 2).

The test consisted of two stages:

- In the first stage, anodic excitation of the sample to be evaluated was carried out at 800 mV vs. SCE (saturated calomel electrode) for 10 s;
- In the second stage, the potentiost at imposed the abandon potential value for 15 min. As a result, the current variation was plotted as a function of time for an imposed potential.

If the current recorded stayed within the cathodic domain (negative values), a fresh measurement cycle was started: excitation for 10 s at 800 mV and current measurement for a potential set at E_abandon_ +25 mV (ASTM recommends +50 mV). The cycles were repeated, each time at a higher potential, until the current measured moved into the anodic domain (positive values). As a result, the crevice potential was determined, which corresponded to the last-but-one measurement for which the current was positive. Once the Teflon ring was removed, a groove of crevice corrosion was noted on the surface of the metal sample.

### 2.4. Intergranular Corrosion

This type of corrosion occurs preferentially at grain boundaries and may be due to the presence of precipitates.

For assessing the intergranular corrosion morphology, chemical and electrochemical tests may be used. Two examples of evaluating intergranular corrosion susceptibility of austenitic steel tubes are being presented:

– Chemical evaluation of tubes #3, 1.4435/316L used in medical devices and endoscopic applications;
– Electrochemical evaluation of tubes #1, 1.44306/304L used in medical devices and endoscopes applications.

#### 2.4.1. Chemical Tests

The standardized tests [36,37,38] to assess the sensitivity of stainless steel to intergranular corrosion, according to ASTM A262-15 [36], have been summarized in Table 4.

Evaluation of intergranular corrosion according to standard ASTM A262-15-the Strauss test [36].

The test medium was a solution of copper sulphate-50% sulfuric acid in the presence of metallic copper, brought to the boiling point (125 °C) for 120 h. This test investigated the intergranular corrosion behavior of steel in the potential range between 110–350 mV. The test setup was in accordance with ASTM A262-15 [36]. The samples to be tested, tubes of #3, 1.4435/316L steel, were placed in specific glass cradle.

#### 2.4.2. Electrochemical Tests

Electrochemical Potentiodynamic Reactivation (EPR) measurements, single or double loop, are methods of examining and assessing the corrosion sensitivity of austenitic steels [37,38,39,40,41,42,43,44,45,46,47].

**Figure 3:**
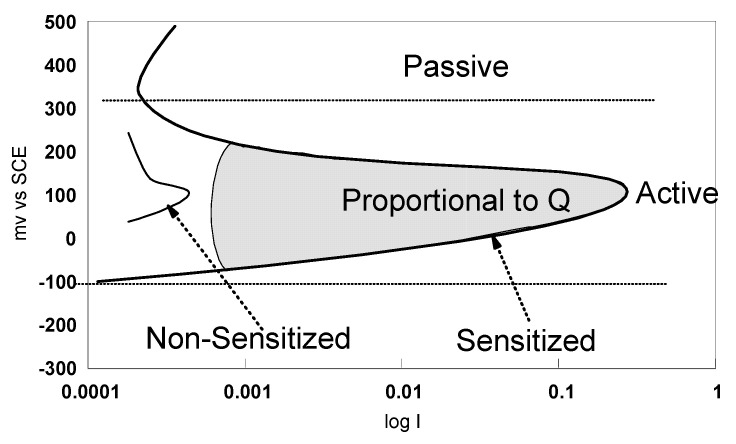
Procedures of single loop EPR test method according to ASTM G108–94 (2015) [48].

The ASTM G108-94 (2015) [48] was practiced with the single loop method. Using the single loop test, a sample polished to a 1 µm finish, was polarized for two minutes at 200 mV vs. SCE in a solution of 0.5 M H_2_SO_4_ + 0.01 M KSCN. Subsequently, the potential was decreased, at a rate of 6 V/h, to the corrosion potential, E_corr_. This decrease resulted in reactivation of the specimen, involving breakdown of the passive film covering chromium depleted areas of material. The area under the large loop generated in the curve of potential vs. current (Figure 3) was proportional to the electric charge Q, that depends on surface area and grain size. In non-sensitized material, the passive film was intact and the loop size was small.

The samples tested were tubes of 304L AISI, used for manufacturing medical endoscopes (Table 5). The tubes #Test 1 and #Test 2 were suspected to be sensitized in intergranular corrosion. For the evaluation test, reference samples #Brut 1 and #Brut 2 and three samples from the supplier stock, subjected to heat treatments at 500, 620 and 750 °C, respectively, were used (Table 5).

The sample (Table 5), transversely cut, was incorporated into a resin and “mirror” polished. The resin was machined to be adapted to the working electrode. The mounting of the electrodes, the electrochemical cell and the EPR measurement conditions were those of ASTM G108-94 (2015) [48]. The ASTM G108-94 method allows a quantitative evaluation of the intergranular corrosion sensitization of steels AISI 304 and AISI 304L. The purpose of the test was to evaluate the intergranular corrosion of the transverse surface by an electrochemical scanning method from +200 mV to −400 mV vs. SCE. In the mathematical calculation for evaluating the sensitivity to intergranular corrosion, another parameter, the corresponding value of the grain index, has to be considered, according to ASTM E112-13 [49].

### 2.5. Galvanic Corrosion

In the first stage, to establish a galvanic series in artificial sweat the open circuit potentials of eighteen alloys were measured:

Precious metal alloys used in jewelry: Pt 950CoNi (950‰Pt, 18‰Co, 32‰Ni), AuPdCu150 (750‰Au, 100‰Pd, 150‰Cu), AuAgNi109 (750‰Au, 141‰Ag, 109‰Ni), AuCuNi130 (750‰Au, 120‰Cu, 130‰Ni), AuNiCu142 (750‰Au, 108‰ Ni, 142‰Cu), AuNiCu112 (750‰Au, 138‰Ni, 112‰Cu) and AuCuZn374 (585‰Au 41‰Cu, 374‰Zn).

Steels: 1.4441, 1.4435, 316 L F, 316 L F Cu, 1.4301, 1.4305, Sandvik 1802, 1.4104. 1.4105, 1.4539 and 12/12.

The samples, in form of 10-mm-diameter discs, were “mirror” polished, washed with a mixture of acetone and ethanol, and rinsed with deionized water 18 MΩ·cm. After drying with hot air, the samples were introduced into the PTFE sample holder, specially designed for the rotating electrode test. The electrochemical measurements were made with a potentiostatic assembly of three electrodes: a working electrode (rotating electrode), a platinum counter-electrode and a reference SCE electrode. Given that diffusion phenomena play a major role with regard to the changes produced at the metal/solution interface and consequently to the state and composition of the metal surfaces layers, readings were made in a laminar system (criterion of Re = 3200) with a limit current iL = 56 mA, and a rotational velocity of 300 rpm, to control the mass transfer phenomena. The open circuit potentials (E_oc_) were measured after 24 h of immersion.

**Figure 4:**
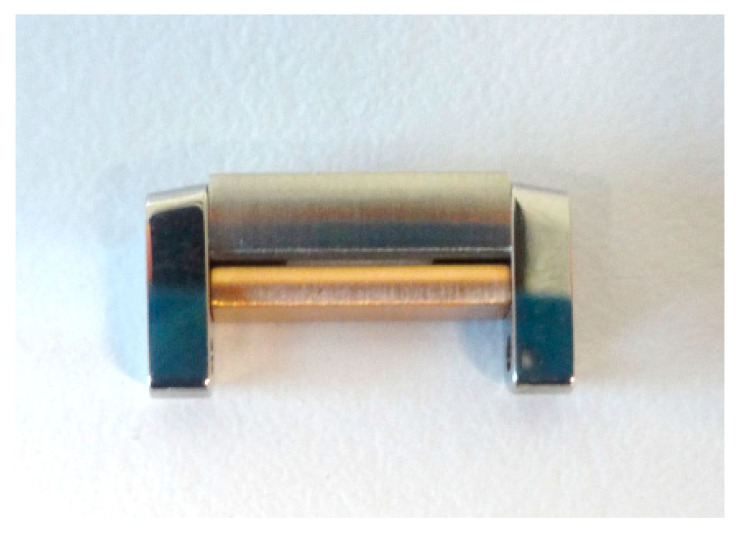
Link, steel-gold assembly with pins.

In the second stage, our interest was focused on galvanic couplings in the assembly of steel watch strap links with precious metal alloys (18K gold). The evaluation was indirectly made, by measuring the quantities of nickel released after 7 days of immersion in artificial sweat, according to standard EN 1811-2011+A1:2015 [50]. The tests were carried out on 27 gold-steel links (Figure 4), 5N18 (18K gold alloy)-1.4441 (316L) and 5N18 (18K alloy)-1.4539 (904L).

The microscopy investigations (scanning electron microscopy/energy-dispersive X-ray spectroscopy SEM/EDX) were carried out using a JEOL JSM-6300 SEM (JEOL, Peabody, MA, USA) equipped with an Oxford INCA EDS system (Oxford Instruments, Abingdon, UK) for local phase analysis.

## 3. Results and Discussion

### 3.1. Uniform Corrosion

**Figure 5:**
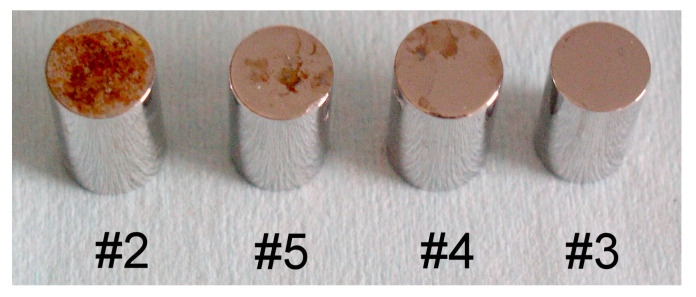
Uniform corrosion salt fog test, according to ASTM B 117-97. Samples #2-1.4427 So, #3-1.4435/316L, #4-316LUgim and #5-316L Val.

After 12 days, the salt fog test revealed that here was a difference in the corrosion susceptibility of transverse and longitudinal surfaces (Figure 5). In general, the transversal surfaces were corroded, with the exception of steel #3, the intensity depending on the steel grade and the manufacturing process. Samples #3, #4 and #5 were made of grade 316L, from three different steelmakers (Germany, France, Italy). The longitudinal surfaces showed no signs of corrosion.

According to Zanotto et al., the test has limitations, it becomes non-discriminating in case of steels with excellent corrosion resistance [51].

**Figure 6:**
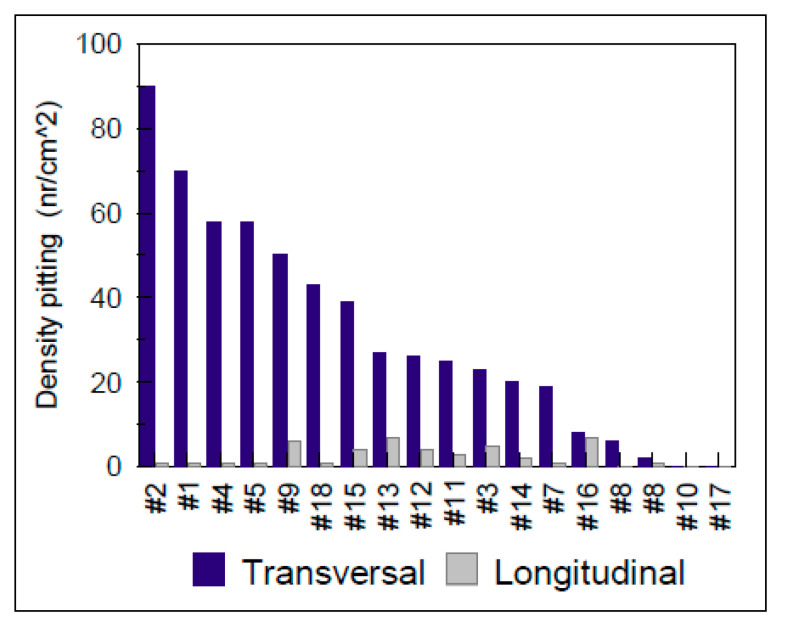
Pitting test results of the transverse and longitudinal surfaces for various grades of steel alloys (0.5 M FeCl_3_ test medium at 50° for 2 h).

In case of steels #8, #10, #6 and #17, the test, carried out under the same experimental conditions, showed no signs of corrosion of the transversal or longitudinal surfaces, similar to sample #3 (Figure 6).

The results obtained are presented in Table 6. In general, the transverse surfaces were corroded with the exception of steel #3. Examination of steel #3 did not reveal any traces of corrosion either in the cold-worked state.

### 3.2. Pitting Corrosion

**Figure 7:**
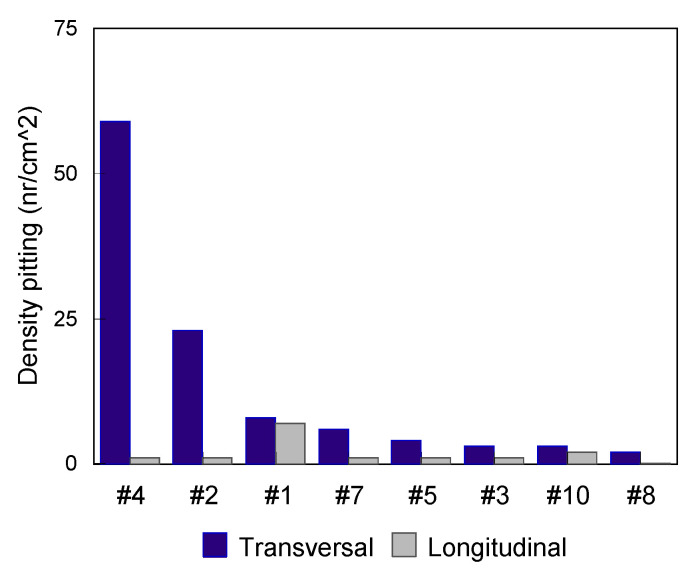
Pitting test results of the transverse and longitudinal surfaces for various grades of steels (0.1 M FeCl_3_ test medium at 37 °C for 15 days).

**Figure 8:**
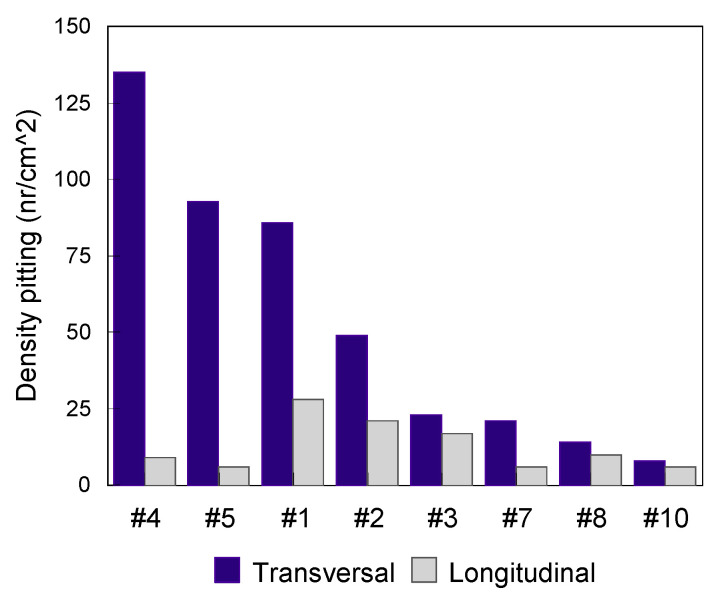
Pitting test results of the transverse and longitudinal surfaces for various grades of steels (0.5 M NaCl test medium at 37 °C for 26 days).

**Figure 9:**
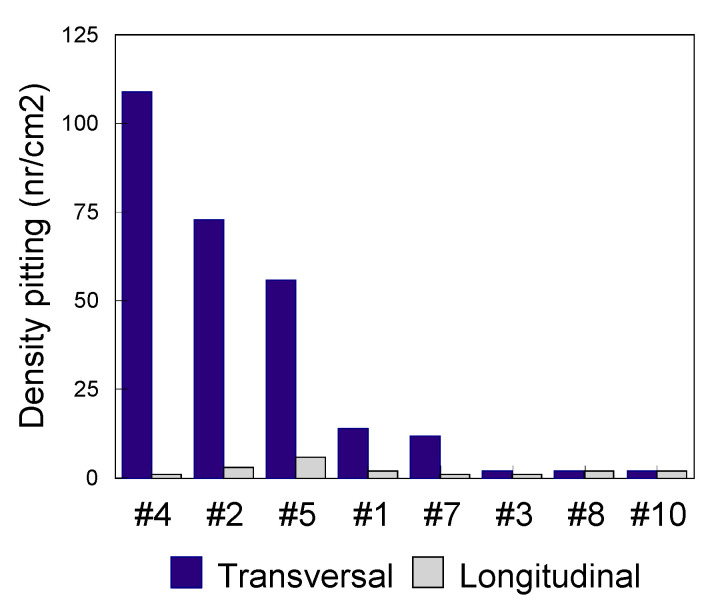
Pitting test results of the transverse and longitudinal surfaces for various grades of steels (artificial sweat test medium at 37 °C for 30 days).

For the evaluation of steels sensitivity in pitting corrosion, available standards allow characterization of the formation, the shapes and the density of pits per unit area [52]. For the establishment of Figure 6, Figure 7, Figure 8 and Figure 9, it was necessary to determine the density of pitting corrosion (counting the number of pits per unit area).

In all test environments, the transverse surfaces showed a higher pitting density compared to the longitudinal ones. 

**Figure 10:**
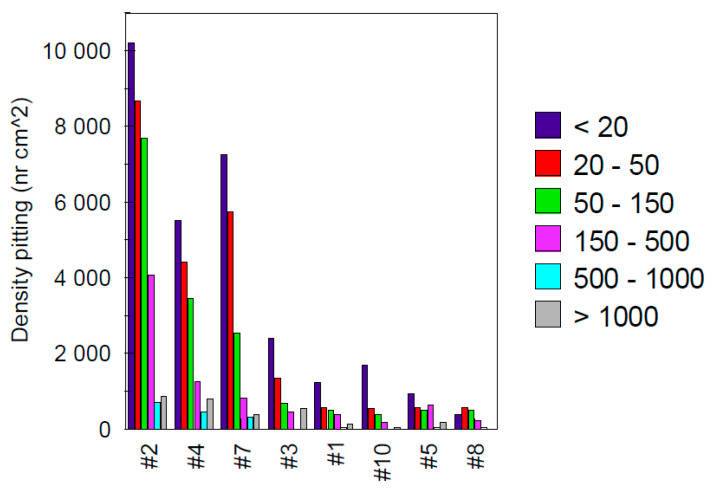
Number of pits counted by class, according to the size of the pit surface area.

Using a Kontron KS 300 Version 1.2 image analysis program, the cross-sectional area of alloys #1, #2, #3, #4, #5, #7, #8 and #10, tested in 0.5 M FeCl_3_ at 50 °C for 2 h was analyzed statistically in relation to the area size of the pits. The following classes were established accordingly: <20, 20–50, 50–150, 150–500, 500–1000 and >1000 μm^2^. The densities (number of pits/cm^2^) according to the above mentioned classes are presented in Figure 10.

The image analysis revealed a density of pits which can be significant (more than 10,000 for sample #2, Figure 10). Under these conditions, it was necessary to define criteria which enable easier identification of pitting corrosion. Examination of the surfaces revealed numerous cavities which were not necessarily pitting.

**Figure 11:**
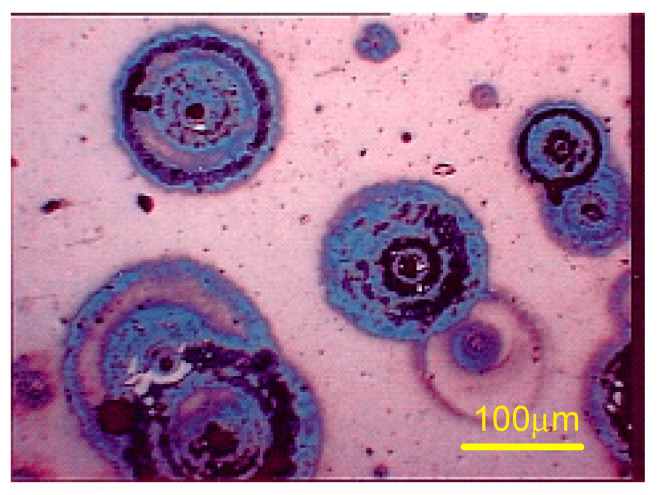
Corrosion pits revealed as blue discs (cross section sample #2).

At this point, it is essential to clarify the definition of pitting corrosion. According to the ASTM, a pitting corrosion is electrochemically active if an anodic dissolution of the alloy occurs within the cavity. Thus, by definition, a pitting corrosion releases cations in the electrolyte. It is therefore sufficient to carry out the corrosion test in an electrolyte containing traces of an analytical reagent which forms, with one of the cations released by the active corrosion pits, an insoluble colored compound which will deposit near the pit. The common element for all the alloys in question is iron, the iron dissolution mechanism being based on ferrous ions (Fe^2+^). Tests carried out on several reagents showed that potassium ferricyanide (K_3_[Fe (CN_6_]) is suitable to form an insoluble colored complex. Corrosion pits, revealed as blue discs encircling the pits (Figure 11), were used to determine the number per unit area.

In conclusion, the FeCl_3_ solution, according to the ASTM G48-11 [31] standard, was aggressive with respect to the pitting corrosion behavior of the alloys studied; within two hours, most steel grades show readily identifiable macroscopic corrosion pits. On the other hand, in case of NaCl 0.5 M or artificial sweat ISO 3160-2 [30], the evaluation of the pits density was problematic given the difficulty of identifying the effectively electrochemically active pits. The use of potassium ferricyanide, which precipitates in the form of Turnbull blue in the presence of Fe^2+^ ions, greatly facilitated the evaluation of the density of pits after immersion in this type of electrolyte.

According to Blackwood [2], pitting corrosion was a common problem with the early 304 stainless steels. In the case of 316L stainless steels, the addition of 2–3 wt% Mo has greatly reduced the number of failures due to pitting corrosion [2]. 

After initiation, pits either keep growing or repassivation may occur. According to Virtanen, an alloy with a high pitting corrosion resistance should ideally combine low susceptibility to pit initiation, low pit propagation rate, and fast repassivation [53]. According to Melchers, in case of metals with electron-conducting passive films such as stainless steels, the number of pits usually correlates inversely with their average depth, since the cathodic current consumed by the large passive surface area fosters anodic dissolution inside the pits [54]. The pits may grow at different rates, depending on the number of active pits [54]. According to Abbasi Aghuy et al., changes in electrochemical behavior of metal due to grain refinement as a consequence of changing grain boundary densities may occur [55].

### 3.3. Crevice Corrosion 

**Figure 12:**
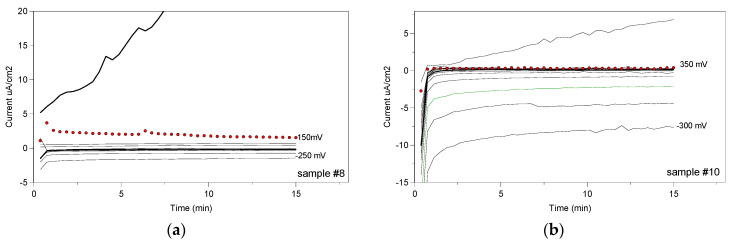
The crevice test potentiostatic curves for the longitudinal surface of sample #8 (**a**) and #10 (**b**).

Figure 12 shows the “variations of the current” curves as a function of time, for values of imposed potential, for the longitudinal surface of samples #8 and #10, respectively. The crevice potentials were +150 mV (red points) for sample #8 and +350 mV (red points) for sample #10.

**Figure 13:**
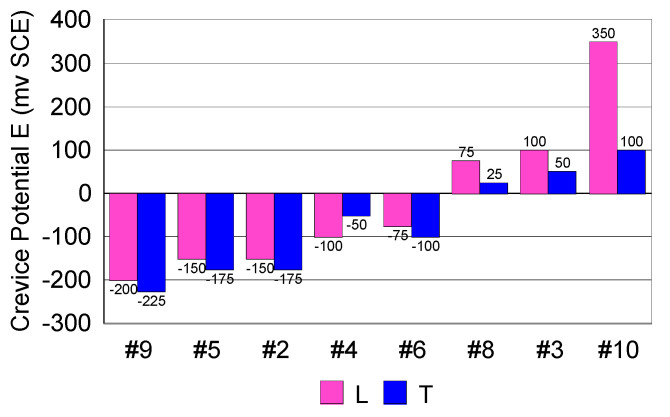
Crevice potential values measured for the transverse and longitudinal surfaces of the steels considered (L: longitudinal, T: transverse).

Figure 13 shows the values of the crevice potentials determined for both longitudinal and transverse surfaces, according to ASTM F746-87 standard [35].

The values of the crevice potentials measured did not reveal any difference in susceptibility to crevice corrosion between the two surfaces. The only difference was that the transverse surface showed lower values than the longitudinal surface, but these differences remained in the field of experimental errors. In other words, there was no significant difference in the crevice corrosion behavior between the two surfaces.

In case of 316L steels (#2, #3, #4, #6, #8, and #9), the values of the crevice potentials were different, due to the structure type of inclusions and composition in minor chemical elements.

The study of Poyetet et al., involving 18-10 type stainless steels [56], has concluded that the reactivity of the inclusions, in terms of their contribution to the onset of pitting, is a function of their association (Table 7).

Mixed oxide-sulfide or silicate-sulfide inclusions are the most susceptible to pitting. The corrosion susceptibility of inclusions might be ranked, in increasing order: sulfides < alumina-sulfides < silicate-sulfides < Mg-oxide-sulfides. By themselves, sulfides do not have a particularly detrimental action on the pitting corrosion resistance of steel, but they become particularly harmful when associated in the form of mixed inclusions. As far as shape is concerned, globular inclusions (present only in the as-cast, undeformed material) seem to be less harmful than inclusions deformed during hot working of the metal [56].

**Figure 14:**
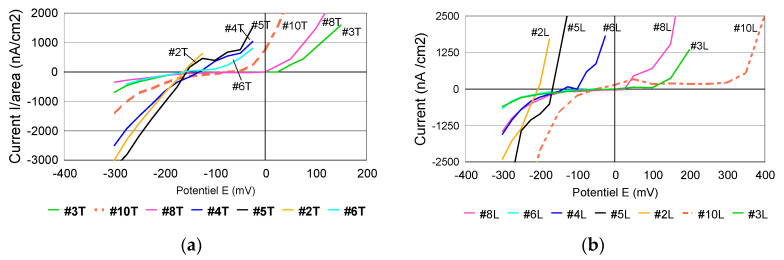
Polarization curves (current value recorded after 15 min vs. preselected potential; (**a**) transverse and (**b**) longitudinal surface.

When considering the final values of currents recorded after 15 min for each level and representing the current as a function of potential, a series of “polarization curves”, specific to the crevice corrosion process were obtained (Figure 14).

When comparing the crevice corrosion behavior of the two surfaces, no real difference in susceptibility to corrosion was noticed. On the other hand, in accordance to Bryant et al. [57], each steel has a different behavior to crevice corrosion. Some steels do not reveal a “passivation capacity” before reaching the value of crevice initiation potential. In case of 316L, respectively #2, #3, #4, #6 and #8 this difference was noticed. According to Liu et al., in case of 316L stainless steel, widely used as a metallic biomaterial, crevice corrosion has been a serious concern [58]. In case of #10, a difference was expected, due to the better corrosion resistance compared to the 316L family.

**Figure 15:**
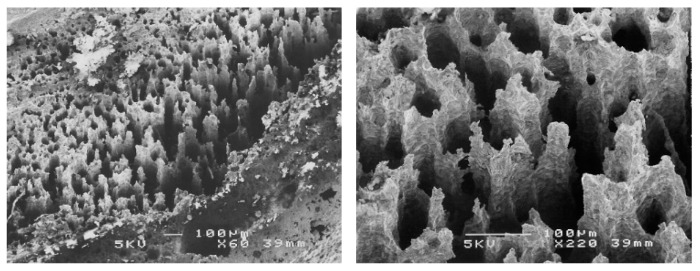
Scanning electron microscopy (SEM) of the transverse surface, corroded under a defective PTFE collar. The columnar morphology suggests preferential longitudinal dissolution due to the texture of the material.

In conclusion, the evaluation of the crevice corrosion resistance did not reveal marked differences between the behavior of the transverse surface compared to the longitudinal one. This shows that the pitting and crevice corrosion mechanisms, although having some similarities, are different. In case of certain grades of steel, particularly sensitive to crevice corrosion, sometimes crevice corrosion can interfere with pitting corrosion measurements. Figure 15 shows a situation where crevice corrosion strongly interfered during the measurement of pitting corrosion by the rotating electrode technique. The crevice corrosion developed under a defective collar, making the measurements unusable for the characterization of pitting corrosion. Sometimes, the observation of the corrosion morphology provides information on the metallographic structure of the alloy. The morphology of crevice corrosion on the transverse surface (Figure 15) showed a particular structure, the orientation of the corroded structures suggesting a preferential longitudinal dissolution. This reveals a manifestation of a higher corrosion sensitivity of the transverse direction compared to longitudinal direction.

### 3.4. Intergranular Corrosion

#### 3.4.1. Chemical Tests

**Figure 16:**
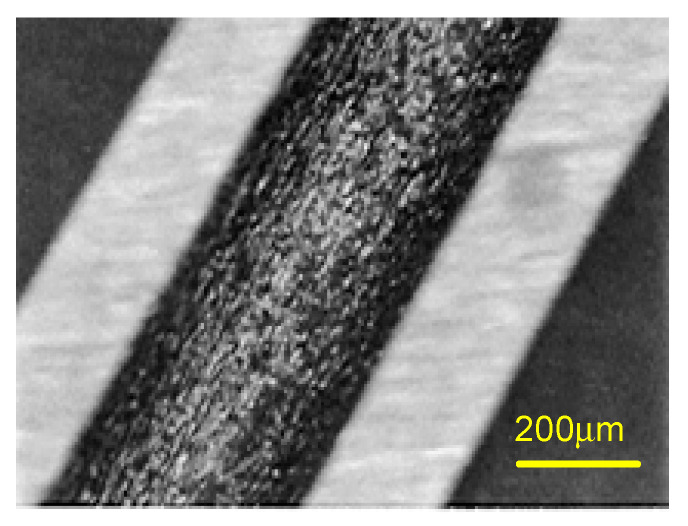
The interior of a corroded tube (#3, 1.4435/316L steel).

**Figure 17:**
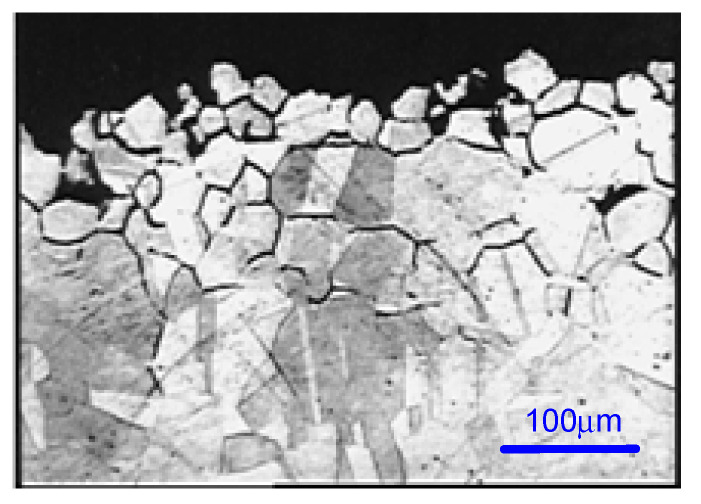
Metallographic section of the tube (#3, 1.4435/316L steel) after Strauss’s test.

After completing the test, the examination of the interior of the tube showed corrosion signs. In case of each sample, a mass loss of about 40 mg/cm^2^ was determined (Table 8). An optical or scanning electron microscopy (SEM) examination was also carried out, for each sample (Figure 16). The metallographic section from the external part of the corroded tube had a structurally disturbed surface area to a depth of about 70 μm (Figure 17).

The Strauss test clearly and unequivocally showed that the 316L steel tubes were sensitized to intergranular corrosion. The corrosion rate was higher on the interior compared to the exterior.

The energy-dispersive X-ray spectroscopy (EDX) analysis of the corroded areas showed the presence of elements which did not belong to the alloy: sulfur, chlorine, calcium, sodium, aluminum and potassium. These elements most likely resulted from lubricants formulated as additives or base oil. The sensitization to intergranular corrosion was probably due to the pyrolysis of residual oil present on the tube surface—in other words, poor cleaning during the manufacturing process.

#### 3.4.2. Electrochemical Tests: EPR Method ASTM G108–94 (2015) 

**Figure 18:**
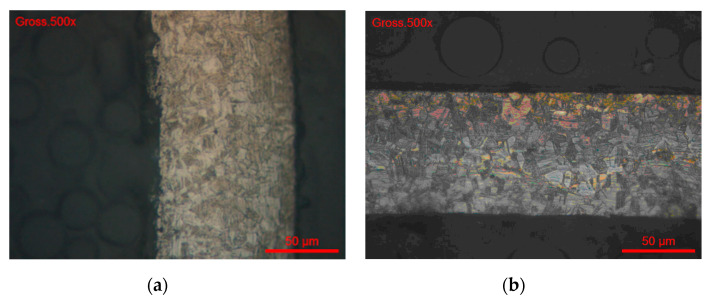
(**a**)#Test 1 and (**b**) #Test 2. Grain index (ASTM E112-13) G = 11.

**Figure 19:**
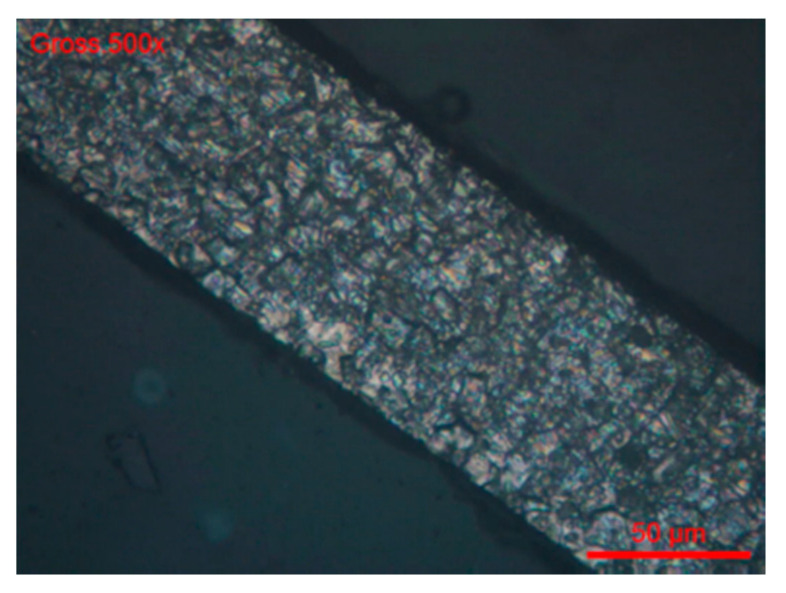
#Brut 1. Grain index (ASTM E112-13) G = 11.

Electrochemical Tests were carried out according to the EPR Method ASTM G108–94 (2015) [48]. The grain index was determined according to the ASTM E112-13 method [49] (Figure 18 and Figure 19).

Grain index values are given in Table 9.

According to the EPR method ASTM G108-94 (2015) [48], after the cyclic polarization scans, the evaluation parameter is the normalized charge (Pa), measured in coulombs/cm^2^, calculated with the formula:

P_a_ = Q/X (1)

where Q = measured on current integration measuring instrument (coulombs), normalized for both specimen size and grain size X = A_s_[5.1 × 10^−3^*e*^0.35G^], where A_s_ = specimen area (cm^2^), G = grain index at 100× according to ASTM E112-13 [49].

In the derivation of the equation, it was assumed that the Q value was the result of the attack on the specimen surface that was distributed uniformly over the entire grain boundary region of a constant width of 2 × (5 × 10^−5^) cm. This may not represent the actual physical processes.

The potentiokinetic electrochemical reactivation results are presented in Table 10.

**Figure 20:**
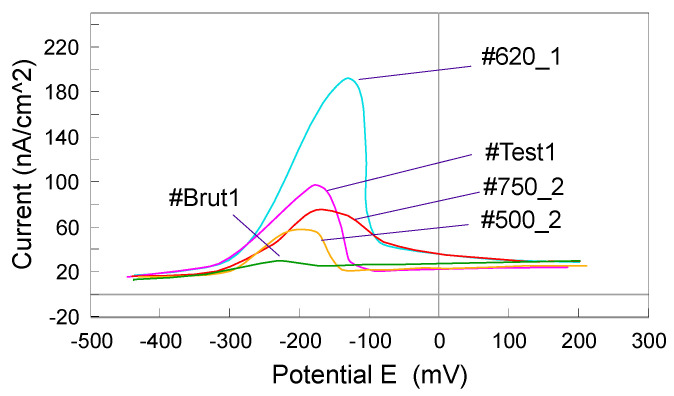
Potentio kinetic reactivation curves recorded for #Brut 1, #Test 1, #500_1, #620_2, and #750_2.

Figure 20 shows the potentio kinetic reactivation curves in linear axes.

**Figure 21:**
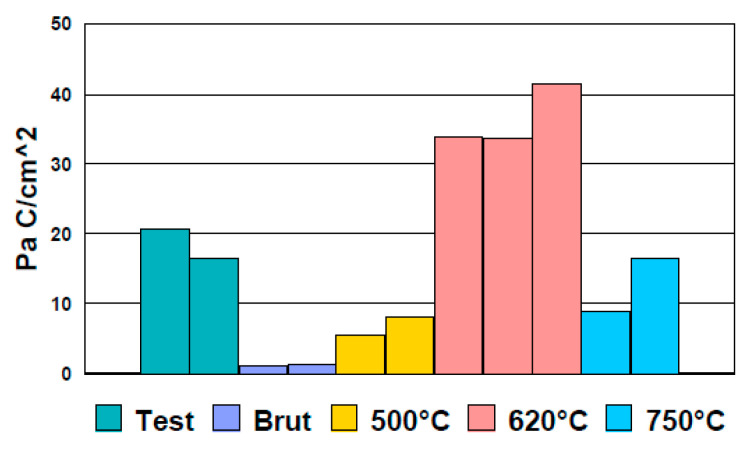
Normalized charge (Pa) measured by EPR.

The peak valuesfor I_r_, given in Table 10, were specific to the intergranular corrosion degradation of the tubes. The higher the intensity, the greater the degradation. Thus, according to Figure 20, it was noted that the highest sensitization of the tubes was generated by the heat treatment at 620 °C. The minimum sensitization corresponded to the 500 °C heat treatment. The overall results (Table 10) for the normalized charge (Pa) calculated (Figure 21) for all the samples indicated that the heat treatment over 500 °C for 304 steels is not indicated, the risks of inducing an intergranular corrosion process being obvious. Consequently, the 500 °C heat treatment should be used in the manufacturing process.

Type 304 steel was more sensitive to intergranular corrosion compared to other steels. Consequently, in the manufacturing process, great importance must be given to this type of corrosion morphology. The temperature of 620 °C was critical for generating the process and therefore used in the ASTM tests (A262-15 and G108-94) for evaluating intergranular corrosion. The goal is to near the behavior of the tube in raw state (#Brut 1).

The susceptibility to intergranular corrosion of stainless steels is not always due to heat treatment with precipitation of chromium carbides. Under certain conditions, the precipitation of intermetallic compounds of (Fe, Cr)Mo_2_ or (Cr, Ni, Fe)_3_P_2_ type can occur. According to Stonawská et al., the structural sensitization of 316 L steel is due to the precipitation of secondary phases along the grain boundaries [59]. The studies of Liu et al. regarding 316 L steels [60] and Fujii et al. [61] regarding 304 steel, also supports this statement. According to Liu et al. [62], the chromium-depleted zones near grain boundaries represent the corrosion nucleation sites for austenitic steels. According to Eliaz, since the carbon content in 316 stainless steel was lowered in the 316L and 316LVM grades, sensitization of this steel is less problematic as it used to be [7].

### 3.5. Galvanic Corrosion

Table 11 presents the corrosion potentials measured in artificial sweat (EN 1811-2011+A1:2015) [50] for the precious alloys and austenitic steels. The measured potential values enable to establish a relative comparison of the alloys nobility in the considered medium and to construct a galvanic series. Higher potential values mean higher corrosion resistance. The alloys series with higher negative potentials (anodic) generally tend to undergo greater corrosion in the event of a galvanic coupling, while other metals (cathodic) will generally undergo a reduced attack. According to Mansfeld and Kenkel, the corrosion potential of each alloy is a criterion in the analysis of galvanic corrosion behavior, but it is still insufficient. The electrical potential values can only indicate a trend and state absolutely nothing about the rate of corrosion and the type of control of the galvanic cell (mixed, cathodic or anodic) [63,64].

The most frequent cases we encountered are precious metal-austenitic steel assemblies in watch straps. Thus millions of gold-steel links are produced to assemble straps, this is the ideal case for the formation of a galvanic cell, a significant difference in electrical potential being involved. In the case of gold-steel, a difference in electrical potential of around 300 mV can be calculated.

According to Gilbert and Mali, while corrosion per se may not be of great concern, when combined with mechanical effects, restricted crevice-like geometries or any combination thereof, considerably amplified corrosion rates might arise [65]. 

**Figure 22:**
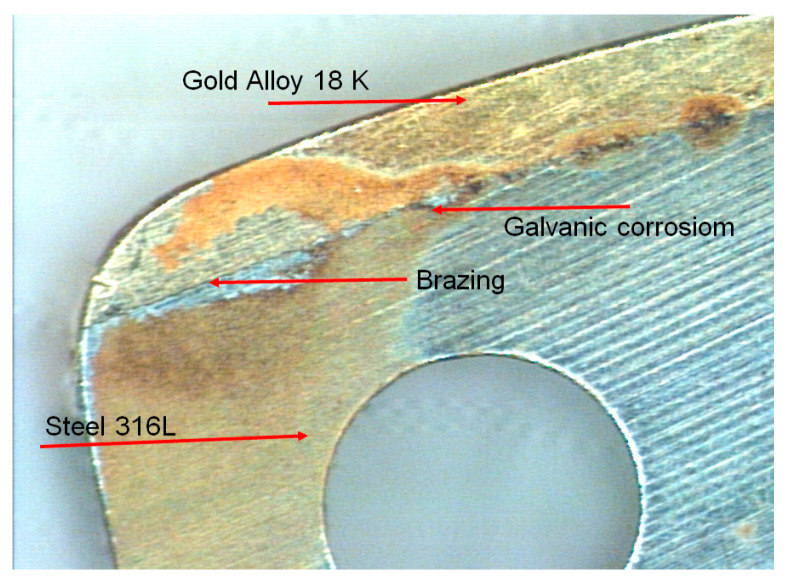
Galvanic corrosion in a gold-steel assembly.

**Figure 23:**
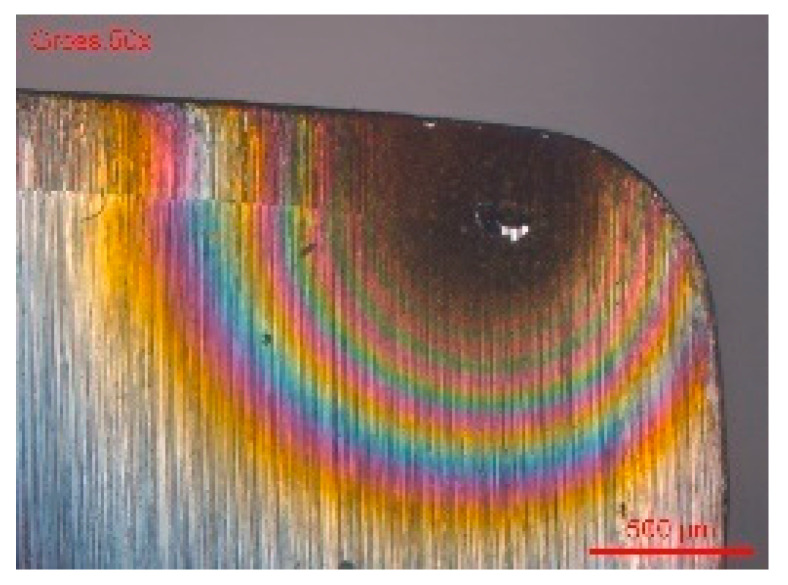
Corrosion of the transverse surface, at the level of the gold-steel interface.

One of the several available techniques to realize the gold-steel assembly is brazed gold caps. The brazed gold caps reveal a particularity due to brazing. The solder acts as the anode (a small area) and the steel and gold parts are the cathode (large area). Thus, the corrosion process results in the dissolution of the solder (Figure 22 and Figure 23). In such a type of assembly it is particularly important to make the right choice of solder.

Two aspects of great importance have to be considered:

(a) The cathode–anode relationship. The precious metal surfaces will act as the cathode, and the less noble parts will be the anode. Constructions with large cathode surfaces and small anode surfaces are very dangerous. The galvanic cell will output a strong anodic current which will lead to the rapid degradation of the anodic part by mechanisms of crevice or pitting corrosion;
(b) The nickel release in contact with the skin has to be considered as the current legislation tolerates an amount of 0.5 µg/cm^2^·week.

Table 12 presents a series of tests carried out on the same types of gold-stainless steel links. The 14441/316 LM steel originated from five different steelmakers from the EU, USA, Japan and China.

**Figure 24:**
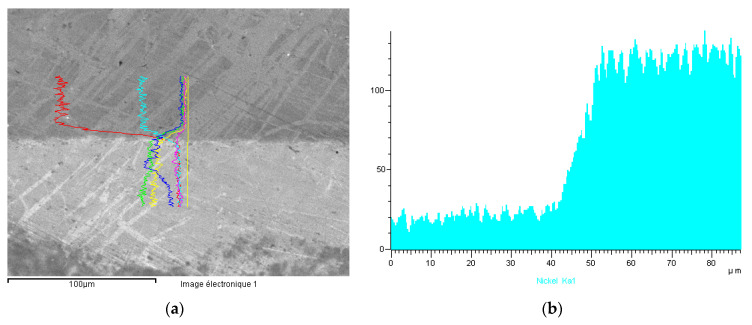
Sample #6. (**a**) EDX profiles of the gold-904 L steel solder for iron, nickel, silver, gold, copper and zinc; (**b**) EDX Ni profile.

**Figure 25:**
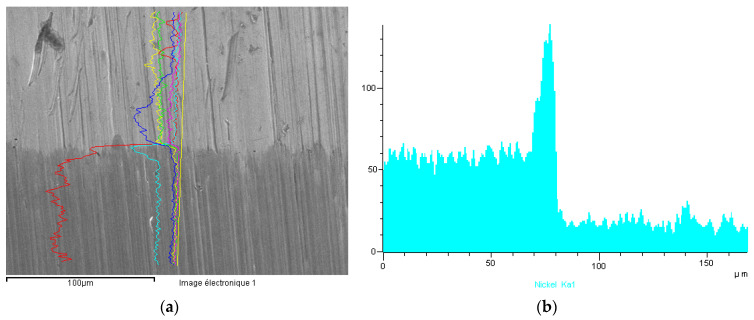
Sample #4. (**a**) EDX profiles of the gold-316 LM steel solder for iron, nickel, silver, gold, copper and zinc; (**b**) EDX Ni profile.

In case of #6 (gold-steel 904L), the nickel release was greater than 2 μg·cm^−2^·week^−1^, despite the absence of visible corrosion. This was due to a different behavior compared to a medical 316L steel. The comparison of the EDX profile between a gold-904L steel (Figure 24) and a gold-316LM steel (Figure 25) revealed a very different nickel profile with the disappearance of the nickel peak in the gold-904L steel solder (Figure 24). In the gold-904L system, the solder was in the anodic position (gold and 904L steel being cathodic), with a very unfavorable surface report. It revealed a selective corrosion morphology powered by a galvanic battery; this would explain the significant nickel release from the gold-904L steel coupling, despite the absence of visible corrosion.

**Figure 26:**
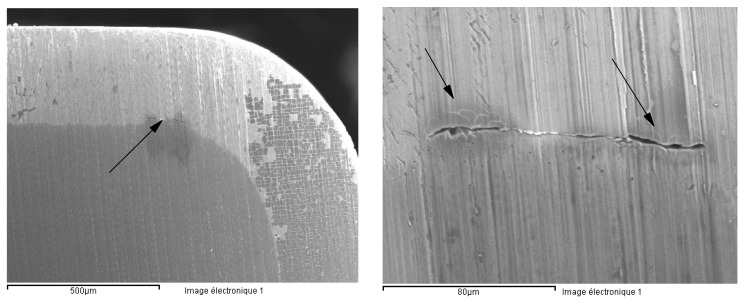
SEM examination of sample #4.

SEM examination of sample #4 (5N18) showed that the corrosion was localized and did not develop at the level of the steel, but of the brazing, causing its dissolution (Figure 26). This demonstrates that the solder represents the weak point of the gold-steel assembly.

The steels which are being used for watch straps are of grades 316 and 904L. The other steel grades, such as 304, 304L, 316LS, and 316Ti, are not usable; their rate of Ni release does not respect the limits imposed by the EU directives, or other countries legislations (USA, Japan, China, Korea, Canada). The difficulty consists in eliminating the corrosion process and achieving a rate of nickel release which respects the legislation: max 0.5 μg·cm^−2^·week^−1^.

The use of a Ni-Cr-P solder involves the risk of increasing the nickel amounts by chemical dissolution or corrosion of the solder. In the case of a gold base solder (melting range 750–850 °C) the risk is to initiate corrosion in the steel; with a Ni-Cr-P based solder (melting range 800–950 °C) the risk is to start corrosion in the solder. To find the best compromise, testing the link assemblies is necessary. Because the quality of 316 LM steel is highly dependent on the supplier, most straps manufacturers use steels they have exclusivity for.

### 3.6. Nickel Release in Relation with the Manufacturing Process

**Figure 27:**
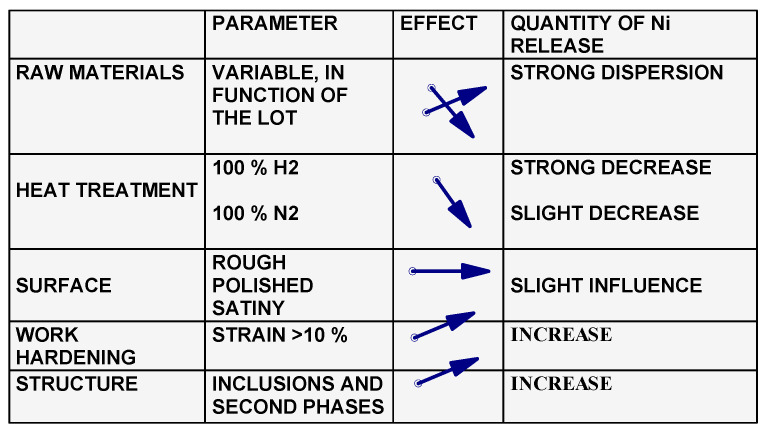
Factors influencing the amounts of nickel released during the manufacturing process.

In our laboratories, a large number of nickel extraction tests in compliance with EN 1811-2011+A1:2015 standard [50] were carried out on the 316 L grades manufactured by five different steelmakers from EU, Japan and USA. There was a significant difference in the quantities of Ni released, compared to the chemical composition of steels, which depends on the steelmaker. The conclusions are presented in Figure 27. It should be pointed out that despite the fact that the grade of steel is in conformity with the classification standards, their behavior was markedly different from one manufacturer to another, due to production parameters, such as the casting volume, alloying additions, and deoxidizing agents.

The heat treatments resulted in a reduction of the nickel release rates. The surface state had little influence. On the other hand, the hardening processes strongly influenced the quantities of nickel released. The increase in hardness greatly decreased the corrosion resistance and increased the amount of nickel released. Another factor which strongly influenced the quantities of nickel released was the inclusion state and the existence of secondary phases in the structure of steels. Being aware of these causes, subcontractors demand from the steelmakers a very strict specification respecting in the manufacturing of steels.

## 4. Conclusions

Seventeen grades of stainless steels were assessed for specific types of corrosion: general, pitting, crevice, intergranular and galvanic. It was noted that there are significant differences between the grades of the austenitic steels studied.

The conclusions are as follows:

– The intensity of the corrosion was dependent on the production parameters, such as the casting volume, alloying additions, and deoxidizing agents;
– The amount of nickel release was dependent on the heat treatment, hardening rate, and other parameters of the manufacturing process;
– The quantity of nickel released is strongly influenced by the inclusion state and the existence of secondary phases;
– There is a clear difference of corrosion between the transverse surface and the longitudinal surface. The longitudinal surface (in the rolling direction) reveals a better resistance to corrosion than the transverse surface.
– The quantities of nickel released are highly dependent on the grade of steel. As a result, manufacturers can use only steels that meet the current legislative requirements;
– Top range watches manufacturers use steels with exclusivity labels, so the chemical compositions, structures, inclusive states, mechanical properties, machinability, polishing are very well defined in their specifications. The rule also applies to medical devices manufacturers;
– Finally, a compromise in choosing a steel over another has to be made, depending on the application and the legal requirements for the final products on a specific market.

## Figures and Tables

**Table 1:** Chemical composition (wt.%) of the grades of austenitic steels used in corrosion tests.

Code	DIN	AISI	C	Si	Mn	P	S	Cr	Mo	Ni	Other
#1	1.4306	304L	<0.03	<1.5	<1.5	<0.035	<0.02	17.0–20.0	-	8.0–12.0	N 0.1–0.2
#2	1.4427So	-	<0.03	<1.0	<2.0	<0.045	0.10–0.13	16.5–18.5	2.0–2.7	-	-
#3	1.4435	316L	<0.03	<1.0	<2.0	<0.045	<0.025	17.0–18.5	2.5–3.0	12.5–15.0	-
#4	1.4435	316LUgim	<0.03	<1.0	<2.0	<0.045	~0.018	17.0–18.5	2.5–3.0	12.5–15.0	N < 0.11
#5	1.4435	316LVal	<0.03	<1.0	<2.0	<0.045	~0.018	17.0–18.5	2.5–3.0	12.5–15.0	N < 0.11
#6	1.4435	316LPM	<0.03	<1.0	<2.0	<0.045	~0.018	17.0–18.5	2.5–3.0	12.5–15.0	N < 0.11
#7	1.4435	316LSW	<0.03	<1.0	<2.0	<0.045	~0.018	17.0–18.5	2.5–3.0	12.5–15.0	N < 0.11
#8	1.4441	316LMed	<0.03	<1.0	<2.0	<0.025	<0.01	17.0–0.19	2.5–3.2	13.0–15.5	N < 0.10 Cu < 0.12
#9	1.4571	316Ti	<0.08	<1.0	<2.0	<0.045	<0.03	16.5–18.5	2.0–2.5	-	-
#10	1.4539	904L	<0.02	<0.7	<2.0	<0.03	<0.015	19.0–21.0	4.0–5.0	24.0–26.0	Cu 1.0–2.0; N 0.04–0.15
#11	1.4057	431	0.14–0.23	<1.0	<1.0	<0.045	<0.03	15.5–17.5	-	1.5–2.5	-
#12	1.4460	329	<0.05	<1.0	<2.0	<0.045	<0.03	25.0–28.0	1.3–2.0	4.5–6.5	N 0.05–0.2
#13	1.4462	2205	<0.03	<1.0	<2.0	-	<0.02	21.0–23.0	2.5–3.5	4.5–6.5	N 0.08–0.2
#14	1.4542	630	<0.07	<1.0	<1.0	<0.045	<0.03	15.0–17.0	-	3.0–5.0	Nb 0.15–0.45
#15	1.4841	310/314	<0.20	1.5–2.5	<2.0	<0.045	<0.03	24.0–26.0	-	19.0–22.0	-
#16	1.4876	B 163	<0.12	<1.0	<2.0	<0.03	<0.02	19.0–23.0	-	30.0–34.0	-
#17	2.4816	Inconel600	<0.15	<0.5	<1.0	<0.02	<0.015	14.0–17.0	-	>72.0	Ti < 0.3; Al < 0.3; B <0.006; Cu <0.5; Fe 6.00–10.0

**Table 2:** The salt fog test conditions.

Test	Conditions
Electrolyte	✓ NaCl ✓ Artificial sweat
Temperature	35 °C
Total duration	12 days
NaCl 5%	6 days
Artificial sweat Dilution x40	6 days
Operating cycle salt spraying exposing	✓ 15 min ✓ 45 min

**Table 3:** Types of electrolytes used and the experimental conditions for testing pitting corrosion [31,32,33].

Test Medium	Concentration	Temperature	Test Duration
FeCl_3_	0.5 M	50 °C	2 h
FeCl_3_	0.1 M	37 °C	15 days
NaCl	0.5 M	37 °C	26 days
Artificial sweat ISO 3160-2 Diluted 20x	NaCl 0.5 g/L; NH4Cl 0.4375 g/L; Acetic Acid 0.063 g/L; Urea 0.125 g/L; Lactic Acid 0.375 g/L; NaOH solid, necessary quantity to induce a pH of 4.7	37 °C	30 days

**Table 4:** ASTM A262-15 [36] Standard practice for detecting susceptibility to intergranular attack in austenitic stainless steels.

Designation	Test	Temperature	Testing Time	Applicability	Evaluation Method
Practice A	Oxalic Acid Etch Screening Test	Ambient	1.5 min	Chromium Carbide sensitization only	Microscopic Examination
B	Ferric Sulfate and 50% Sulfuric Acid	Boiling	120 h	Chromium Carbide	Weight loss/ Corrosion Rate
C	65% Nitric Acid	Boiling	4 h	Chromium Carbide	Weight loss/ Corrosion Rate
D	10% Nitric-3% Hydro Fluoric Acid (This test has been removed from A 262-15)	70 °C	4 h	Chromium carbide in 316, 316L, 317, 317L	Corrosion Rates of “unknown” over that of solution annealed specimen
E	6% Copper Sulfate 16% Sulfuric Acid with metallic copper	Boiling	24 h	Chromium Carbide	Examination for fissures after bending
F	Copper Sulfate 50% Sulfuric Acid with metallic copper	Boiling	120 h	Chromium Carbide in 316 and 316L	Weight loss/ Corrosion rate

**Table 5:** Test samples #1, 14306/304L tubes.

Code	Description
#Test 1	Tube returned from user
#Test 2	Tube returned from user
#Brut 1	Tube from supplier stock (reference)
#Brut 2	Tube from supplier stock (second reference)
#500	Tube from supplier stock+ 500 °C heat treatment, 1 h
#620	Tube from supplier stock+ 621 °C heat treatment, 1 h
#750	Tube from supplier stock+ 750 °C heat treatment, 1 h

**Table 6:** Observations after the salt fog test (samples tested in annealed, cold-worked state).

Code	State	T *-Face	L **-Face
#2	Annealed	Corrosion	No corrosion
	Cold worked	Corrosion	No corrosion
#3	Annealed	No corrosion	No corrosion
	Cold worked	No corrosion	No corrosion
#4	Annealed	Corrosion	No corrosion
	Cold worked	Corrosion	No corrosion
#5	Annealed	Corrosion	No corrosion
	Cold worked	Corrosion	No corrosion

* T: transverse surface, ** L: longitudinal surface, with respect to the rolling direction.

**Table 7:** Types and reactivity of inclusions according to [56].

Inclusion Types and Associations	Number Rating: #Pitted/#Total of Inclusions	Shape Rating: #Pitted/#Total of Inclusions
Sulfide Sulfide-silicate Silicate	2/32 = 6% 22/81 = 27% 0/5 = 0%	Globular 3/20 = 15% Elongated 19/61 = 31%
Sulfide Sulfide-alumina Alumina	1/33 = 3% 9/87 = 10% -	Globular 1/33 = 3% Elongated 8/54 = 15%
Mg-oxide Mg-oxide-sulfide	100%	(rare inclusions)

**Table 8:** Tube mass loss after Strauss test.

Material	Mass Loss (g)	Surface (cm^2^)	Mass Loss (mg/cm^2^)
Tubes 1.4435/316 L	0.31651	7.38	43
	0.32816	7.80	42
	0.28091	7.02	40
	0.22120	5.84	38
	0.05145	6.04	9

**Table 9:** Grain index according to ASTM E112-13 [49].

Code	Grain Index
#Test 1	11
#Test 2	11
#Brut 1	11

**Table 10:** Potentiokinetic electrochemical reactivation results.

Code	E_oc_ (mV)	I_r_ (mA/cm^2^)	Q (C/cm^2^)	P_a_(C/cm^2^)
#Test 1	−400	8.21	4.96	20.67
#Test 2	−423	12.92	4.01	16.70
#Brut 1	−387	11.84	0.28	1.15
#Brut 2	−410	8.21	0.33	1.37
#500_1	−410	30.70	1.33	5.54
#500_2	−388	59.22	1.94	8.10
#620_1	−404	173.30	8.17	34.04
#620_2	−410	132.60	8.07	33.63
#620_3	−395	162.20	9.94	41.42
#750_1	−407	34.22	2.14	8.90
#750_2	−407	59.22	4.01	16.70

E_oc_ = Initial open circuit potential, I_r_ = maximum anodic current density.

**Table 11:** Galvanic series established in an EN1811-2011+A1:2015 artificial sweat type environment.

A	Precious Alloys	Corrosion Potential E_corr_ (mV)	B	Steels	Corrosion Potential E_corr_ (mV)
952	Pt 950CoNi	175	316L series	1.4441	−21
150	AuPdCu150	70	-	1.4435	−160
109	AuAgNi109	53	-	316L F	−164
141	AuCuNi130	43	-	316L F Cu	−282
142	AuNiCu142	42	304 series	1.4301	−169
112	AuNiCu112	22	303 series	1.4305	−266
374	AuCuZn374	6.4	-	Sandvik 1802	−168
-	-	-	-	1.4104	−234
-	-	-	-	1.4105	−389
-	-	-	904L series	1.4539	−72
-	-	-		12/12	−163

**Table 12:** Tests results for gold-stainless steel links.

#	Gold	Steel	Nickel Release (μg·cm^−2^·Week^−1^)	Corroded Parts Rate	Corrosion Rate (%)
#1	5N18	1.4441	0.14	0/6	0%
		316LM	0.25		
			0.13		
			0.04		
			0.22		
			0.03		
#2	5N18	1.4441	0.44	2/6	33%
		316LM	0.05		
			0.01		
			0.10		
#4	5N18	1.4441	0.03	6/9	67%
		316LM	0.06		
			0.09		
#5	5N18	1.4441	0.09	0/3	0%
		316LM	0.08	-	-
			0.06		
#6	5N18	1.4539	2.3	0/3	0%
		904L	2.6		
			2.2		
